# Near-death experiences, attacks by family members, and absence of health care in their home countries affect the quality of life of refugee women in Germany: a multi-region, cross-sectional, gender-sensitive study

**DOI:** 10.1186/s12916-017-1003-5

**Published:** 2018-02-01

**Authors:** Jenny Jesuthasan, Ekin Sönmez, Ingar Abels, Christine Kurmeyer, Jana Gutermann, Renate Kimbel, Antje Krüger, Guenter Niklewski, Kneginja Richter, Ulrich Stangier, Anja Wollny, Ulrike Zier, Sabine Oertelt-Prigione, Meryam Shouler-Ocak, on behalf of the Female Refugee Study (FRS) Investigators

**Affiliations:** 1 Psychiatric University Clinic of Charité at St. Hedwig Hospital, Große Hamburger Straße 5 – 11, 10115 Berlin, Germany; 2 Department of Psychiatry, Marmara University, School of Medicine, Istanbul, Turkey; 3 Office of the Equal Opportunities Officer, Charité-Universitätsmedizin, Berlin, Germany; 4 Department of Clinical Psychology and Psychotherapy, Institute of Psychology, Goethe University Frankfurt, Frankfurt, Germany; 5 Insitute of Occupational, Social and Environmental Medicine, University Medical Center of the University of Mainz, Mainz, Germany; 6 Insitute of General Practice, University Medical Center Rostock, Rostock, Germany; 7 University Clinic for Psychiatry and Psychotherapy, Paracelsus Medical University Nuremberg, Nuremberg, Germany; 8 Faculty for Social Sciences, Technical University Nuremberg GSO, Nuremberg, Germany; 9 Faculty for Medical Sciences, UGD University Stip, Stip, Macedonia; 10 Department of Primary and Community Care, Radboud University Medical Center, P.O. Box 9101, 6500 HB Nijmegen (117), The Netherlands; 11 Institute of Legal Medicine, Charité-Universitätsmedizin, Turmstr. 21, Haus N, 10559 Berlin, Germany

**Keywords:** Refugees, Women, Quality of life, Traumatic experiences, Migration

## Abstract

**Background:**

The year 2016 has marked the highest number of displaced people worldwide on record. A large number of these refugees are women, yet little is known about their specific situation and the hurdles they have to face during their journey. Herein, we investigated whether sociodemographic characteristics and traumatic experiences in the home country and during the flight affected the quality of life of refugee women arriving in Germany in 2015–2016.

**Methods:**

Six hundred sixty-three women from six countries (Afghanistan, Syria, Iran, Iraq, Somalia, and Eritrea) living in shared reception facilities in five distinct German regions were interviewed by native speakers using a structured questionnaire. Sociodemographic data and information about reasons for fleeing, traumatic experiences, symptoms, quality of life, and expectations towards their future were elicited. All information was stored in a central database in Berlin. Descriptive analyses, correlations, and multivariate analyses were performed.

**Results:**

The most frequent reasons cited for fleeing were war, terror, and threat to one’s life or the life of a family member. Eighty-seven percent of women resorted to smugglers to make the journey to Europe, and this significantly correlated to residence in a war zone (odds ratio (OR) = 2.5, 95% confidence interval (CI) = 1.4–4.6, *p* = 0.003) and homelessness prior to fleeing (OR = 2.1, 95% CI = 1–4.3, *p* = 0.04). Overall the described quality of life by the women was moderate (overall mean = 3.23, range of 1–5) and slightly worse than that of European populations (overall mean = 3.68, *p* < 0.0001). The main reasons correlating with lower quality of life were older age, having had a near-death experience, having been attacked by a family member, and absence of health care in case of illness.

**Conclusions:**

Refugee women experience multiple traumatic experiences before and/or during their journey, some of which are gender-specific. These experiences affect the quality of life in their current country of residence and might impact their integration. We encourage the early investigation of these traumatic experiences to rapidly identify women at higher risk and to improve health care for somatic and mental illness.

**Electronic supplementary material:**

The online version of this article (doi:10.1186/s12916-017-1003-5) contains supplementary material, which is available to authorized users.

## Background

Longstanding conflicts in disrupted societies in the Middle East and eastern Africa have forced millions of civilians to flee their countries, leading to the largest migration wave since World War II [1]. According to the United Nations High Commissioner for Refugees (UNHCR), in the year 2016, 65.6 million people were living in an external or internal situation of displacement [2], the highest number since records began in 1951. Refugees flee their home countries due to war, internal conflict, threat to their lives and those of their families, experiences of violence, and inability to provide food and shelter for themselves and their families [3, 4]. The reasons for leaving one’s country are varied and complex, including political, logistic, economic, and social factors [4, 5]. This decision is often taxing and frequently seen as unavoidable, thus representing a major life event for all affected [5–7].

The decision to flee is followed by the journey itself, which in the case of people arriving in Europe can last several years [2]. The flight generally includes travel by land and sea under harrowing conditions and involves extensive physical and psychological stress, leading to a number of health consequences [8]. Refugees experience bodily injuries and illness, mostly related to the travel and housing conditions along the way, as well as frequent psychological trauma. Once they reach their destination, acute physical concerns are treated, but psychological issues are frequently ignored due to logistic and capacity limitations [9–11].

Fleeing and living as a refugee can be traumatic events for everyone. However, gender differences can significantly affect the experience. Women and men as well as girls and boys embody different roles within their societies of origin and are assigned different roles. Women are more frequently less educated than men in their countries of origin and more frequently tend to family and care duties rather than working outside the home [12, 13]. Women are also more vulnerable to all forms of violence in their home countries and during flight, leading to physical abuse and psychological traumatization [14]. The gendered expectations towards their roles might be questioned once they reach their country of destination, where gender roles could differ. This can lead to conflict and self-questioning [15, 16].

Little research is available about the specific situation and flight experiences of women arriving in Germany in 2015–2016 from the Middle East and eastern Africa. We wanted to explore their situation in their home countries, their motivations for fleeing and experiences during their journey, as well as their quality of life, health, and needs perception once they reached the receiving country. Furthermore, we sought to identify personal factors and experiences on their journey associated with improved or worsened quality of life, in order to facilitate the identification of those in major need of support.

## Methods

### Study design and participants

The current study was funded by the Bundeskanzlerinnenamt (German Federal Chancellery) to Charité-Universitätsmedizin and its four regional partners (Rostock for Mecklenburg-Western Pomerania, Mainz for Rhineland-Palatinate, Frankfurt for Hesse, and Nuremberg for Bavaria). The participating institutions represent the regional and structural/legal differences refugees experience in Germany. The study was set up for a duration of 12 months to collect the first representative data on the specific situation of refugee women in Germany. The German Federal Chancellery defined the investigated nationalities, based on the likelihood of successful decision on the asylum application and granting of the refugee status. At the time of the granting process, these countries were Afghanistan, Syria, Iran, Iraq, Somalia, and Eritrea. We contacted the BAMF (Federal Office for Migration and Refugees) to obtain statistical data about the distribution of the refugee population in Germany and calculated quotas for enrollment in each project site. Refugees in Germany are supposed to be equally distributed among the 16 federal states according to a specific statistical mechanism (“Königssteiner Schlüssel” [17]) which takes into account population and the federal states’ fiscal revenue. Based on the national statistics about asylum procedures in the year 2015 (obtained from BAMF), we recruited a total of 663 women, 257 from the city of Berlin, 98 from Hesse, 105 from Mecklenburg-Western Pomerania, 87 from Rhineland-Palatinate, and 116 from Bavaria. Within these regional totals, projected home country distributions were calculated based on the national statistics.

### Recruitment

We recruited women exclusively from shared reception facilities in five German regions. The local project coordinators contacted all reception facilities within the city or regional district. Initial contact was established with the facility management and the study presented. Subsequently internal informational events were organized with the support of the facility management and social workers in the shelters. This was the primary recruitment mode; however, direct informal invitation was also employed in several sites. At the informational event, which lasted approximately one hour and a half, native speakers of Arabic, Dari/Farsi, Somali, and Tigrinya presented the study and information materials to the women living in the shelters who attended the informational event. We then handed out participant’s information sheets in five languages (English, Arabic, Farsi, Somali, and Tigrinya) and invited the women to share their contact information if they were interested in participating. At least 24 hours passed from the provision of the participant’s information to the interview with the prospective participants who gave consent. We met the participants at their reception facilities of residence and administered the questionnaire in a private location in a one-on-one interview. One reception facility could not guarantee a secluded space, so the women were transported to the recruitment center to conduct the interviews. If the women were illiterate, the interviewer read the questions and filled out the questionnaire; if the participants were literate, they filled it out themselves, supported by the interviewer for the open questions. The interviewers were trained to ask a single follow-up question if the women chose to not answer single questions. If the women chose not to respond, the interviewers skipped the question to avoid crossing boundaries and creating any re-traumatization. Interviews lasted between one hour and two and a half hours. All interviewers were native speakers who had obtained training by the team supervisors in interviewing traumatized subjects, providing personal protection from vicarious traumatization, and setting appropriate boundaries. Supervision for interviewers was provided throughout the duration of the study at all recruitment centers.

All project partners sought ethical approval within their institution of reference — university or region, depending on the regional law — and obtained it (reference numbers are: Berlin: EA1/117/16, Nuremberg: 016/1511, Rostock: A2016-0142, Frankfurt a. M.: 334/16, Mainz: 837.316.16 (10635)). All procedures complied with the declaration of Helsinki.

### Questionnaires

The questionnaires were designed to assess the following domains: sociodemographic information, information about experience prior to and during the flight, quality of life and clinical psychological symptoms, experiences in Germany since arrival, health behavior and help-seeking practices, and future wishes and goals. Sociodemographic variables and experiences during the flight were elicited through investigator-designed questions if needed. The traumatic events experienced or witnessed were assessed using the Harvard Trauma Questionnaire (HTQ) [18, 19]. This allows the assessment of traumatic experiences in the population, but it does not allow for a clinical diagnosis of traumatization. Psychological symptomatology was assessed using the Hopkins Symptom Checklist (HSCL-25) [20]. Symptoms of somatization were assessed using the SCL questionnaire, somatization subscales [20]. Quality of life in terms of psychological, physical, social, and environmental well-being was determined using the EUROHIS-QOL questionnaire [21]. Racially based discrimination was assessed in a subgroup of participants using the International Comparative Study of Ethno-cultural Youth (ICSEY) questionnaire [22]. Personal experiences in the shelters in Germany as well as health behavior and health-seeking practices were elicited through open questions. Finally, the women described their wishes and goals in 5 years’ time via open questions. Questionnaires were translated into Arabic, Farsi, Somali, and Tygrinja and translated back into German to ascertain content validity [23]. The full questionnaire was piloted in a set of 15 women of different age, socioeconomic, and educational levels to test feasibility, duration, and comprehension.

### Statistical analysis

Our main hypothesis was that traumatic experiences in the home country and during the flight affected quality of life of the women surveyed. The secondary aim was the identification of sociodemographic and traumatic influencers of quality of life. We computed descriptive statistics to define the overall population structure and identify differences in traumatic experiences among groups. Means of quality of life were compared with European data [24] using *t* tests. In a first step, logistic regression models including sociodemographic explanatory variables were fitted to identify significant associations. Separately, the relationship between trauma and quality of life was also assessed using multivariate regression models adjusted for all traumatic experiences. Missing data were less than 3% for all included variables and, hence, no imputation was calculated. Two-tailed analyses were conducted, and a *p* value < 0.05 was considered statistically significant. In a second step, comprehensive models using age, family status, having children, education and work experience, as well as traumatic experiences on the flight as explanatory variables for quality of life were fitted and reduced progressively by elimination of non-significant (*p* > 0.1) explanatory variables to avoid overfitting. Likelihood ratio tests were performed to confirm redundancy of the eliminated variables. This was conducted until no further reduction was possible. Automated forward and backward regression models were calculated and confirmed the final model. All statistics were performed using Stata version 13 (StataCorp, College Station, TX, USA).

## Results

### Family status, education, and reasons for fleeing differed based on country of origin

The largest subgroup of our study population consisted of women from Syria (47%) followed by those from Afghanistan (25%) and Iraq (11%). The women recruited differed in their family status based on region of origin, Middle East versus eastern Africa (Table 1). While women from Afghanistan, Syria, Iran, and Iraq were mostly married and accompanied by their partners, women from Somalia and Eritrea more frequently indicated their family status as single or widowed and traveled alone to Germany (Additional file 1: Table S1). Of Syrian, Afghani, and Iraqi female refugees, 84–86% reported having children, and most of them accompanied their mothers on the journey. Tragically, five women reported losing a total of eight children on the journey to Europe, mostly due to drowning.

**Table 1:** Sociodemographic description of the study population in their home countries

	Number, *N*	Age group (years)			Family status		Children		Education			Employment	
		17–29	30–45	46–69	Alone	Partnered	Yes	No	None	School/job training	University	Yes	No
Afghanistan	163 (25%)	76 (48%)	68 (42%)	16 (10%)	27 (17%)	135 (83%)	138 (85%)	25 (15%)	52 (32%)	97 (60%)	13 (8%)	102 (63%)	61 (37%)
Syria	312 (47%)	130 (42%)	127 (41%)	55 (18%)	61 (20%)	250 (80%)	257 (84%)	50 (16%)	24 (8%)	231 (75%)	53 (17%)	176 (56%)	136 (44%)
Iraq	73 (11%)	38 (52%)	23 (32%)	12 (16%)	14 (19%)	59 (81%)	62 (86%)	10 (14%)	15 (21%)	46 (63%)	12 (16%)	47 (64%)	26 (36%)
Somalia	20 (3%)	14 (70%)	4 (20%)	2 (10%)	13 (65%)	7 (35%)	12 (67%)	6 (33%)	11 (55%)	9 (45%)	0	13 (65%)	7 (35%)
Iran	38 (6%)	19 (50%)	17 (45%)	2 (5%)	7 (19%)	30 (81%)	21 (57%)	16 (43%)	3 (8%)	22 (59%)	12 (32%)	21 (55%)	17 (45%)
Eritrea	57 (9%)	40 (70%)	13 (23%)	4 (7%)	28 (51%)	27 (49%)	34 (60%)	23 (40%)	3 (5%)	53 (93%)	1 (2%)	42 (74%)	15 (26%)
Total	663 (100%)	317 (48%)	252 (38%)	91 (14%)	150 (23%)	508 (77%)	524 (80%)	130 (20%)	908 (16%)	458 (70%)	91 (14%)	401 (60%)	262 (40%)

The most commonly reported reasons for fleeing were war, terror, and threat to one’s life or the life of the family members (Additional file 2: Table S2). This applied mostly to women originating from regions of open conflict, such as Afghanistan, Syria, and Iraq. Fear of abduction and inability to secure elementary needs were also most commonly reported in these populations. Women originating from Somalia most frequently reported gender-based risks, such as gender-based violence, fear of honor killings, and forced marriage (Additional file 2: Table S2). Women from Syria and Iraq most frequently reported an inability to secure their basic needs, such as food and water, as reasons for fleeing.

### Women frequently resorted to smugglers, and unconditional support on their journey was limited

Eighty-seven percent of the interviewed women reported resorting to smugglers for their journey. Residence in a war zone (odds ratio (OR) = 2.5, 95% confidence interval (CI) = 1.4–4.6, *p* = 0.003) and homelessness before fleeing (OR = 2.1, 95% CI = 1–4.3, *p* = 0.04) significantly correlated with the likelihood of resorting to smugglers. Free support on the journey was limited. Most commonly fellow travelers provided some form of support. Non-governmental organizations were cited as sources of support by 33% of the women from Syria and 26% of the women from Iraq, but seldom by all others. Governmental organizations were mentioned to varying degrees by women from Afghanistan, Syria, and Iraq (Additional file 3: Table S3). Women from Somalia and Eritrea reported much less support of any form during their flight. Women from Syria most frequently reported support in the form of food, clothing, transportation, and other items, followed by women from Iraq and Afghanistan (Additional file 4: Table S4).

### Women personally experienced a significant number of traumatic events in their home countries and during their flight

Fifty-six percent of the interviewed women reported residing or working in an open war zone before fleeing. Homelessness (52%), hunger and thirst (46%), and no access to medical care (36%) were frequent traumatic events (Table 2). Forty-one percent of the interviewed women reported a personal experience of a near-death situation. Traumatic experiences related to close friends and family members were also reported to relevant degrees, such as forced separations (32%), witnessed killings of family members (26%), or unnatural deaths (27%). Torture was reported by 14% of the women, sexual aggression from strangers or family members by 8% and 5%, respectively. Three percent of the women admitted to committing acts of violence or killings themselves.

**Table 2:** Traumatic experiences in home country/during flight

	Experienced myself	Witnessed	Heard about it	None
Natural catastrophe (*n* = 651)	148 (22.7%)	40 (6.1%)	116 (17.8%)	357 (54.8%)
Severe accident (*n* = 655)	194 (29.6%)	145 (22.1%)	157 (24%)	178 (27.2%)
Life-threatening illness (*n* = 652)	73 (11.2%)	125 (19.2%)	113 (17.3%)	347 (53.2%)
Serious injury (*n* = 647)	83 (12.8%)	163 (25.2%)	115 (17.8%)	312 (48%)
Near-death experience (*n* = 649)	265 (40.8%)	109 (16.8%)	88 (13.5%)	216 (33.3%)
Hunger or thirst (*n* = 652)	302 (46.3%)	41 (6.3%)	85 (13.7%)	231 (35.4%)
Homelessness (*n* = 650)	335 (51.5%)	62 (9.5%)	68 (10.5%)	217 (33.4%)
No access to medical care (*n* = 649)	233 (35.9%)	76 (11.7%)	66 (10.2%)	298 (45.9%)
Mission/residence in war zone (*n* = 648)	355 (54.8%)	50 (7.7%)	59 (9.1%)	196 (30.3%)
Aggression from family member (*n* = 652)	121 (18.6%)	42 (6.4%)	57 (8.7%)	439 (67.3%)
Aggression from stranger (*n* = 653)	144 (22.1%)	64 (9.8%)	88 (13.5%)	373 (57%)
Imprisonment (*n* = 649)	85 (13.1%)	64 (9.9%)	145 (22.3%)	357 (55%)
Forced isolation (*n* = 636)	97 (15.3%)	28 (4.4%)	66 (10.4%)	446 (70.1%)
Brain washing (*n* = 641)	28 (4.4%)	10 (1.6%)	59 (9.2%)	546 (85.2%)
Torture (*n* = 647)	91 (14.1%)	52 (8%)	134 (20.7%)	384 (59.4%)
Sexual aggression from family member (*n* = 647)	33 (5.1%)	14 (2.2%)	52 (8%)	548 (84.7%)
Sexual aggression from stranger (*n* = 644)	54 (8.4%)	22 (3.4%)	101 (15.7%)	470 (73%)
Sexual contact as a minor (*n* = 646)	77 (11.9%)	12 (1.9%)	62 (9.6%)	499 (77.2%)
Forced separation from family (*n* = 646)	203 (31.4%)	20 (3.1%)	35 (5.4%)	390 (60.4%)
Killing of family member or friend (*n* = 646)	170 (26.3%)	77 (11.9%)	165 (25.5%)	258 (39.9%)
Unnatural death of family member (*n* = 646)	175 (27.1%)	66 (10.2%)	157 (24.3%)	258 (39.9%)
Killing of one or more strangers (*n* = 641)	78 (12.2%)	98 (15.3%)	234 (36.5%)	244 (38.1%)
Disappearance or kidnapping (*n* = 650)	92 (14.2%)	64 (9.9%)	236 (36.3%)	260 (40%)
Perpetrator of injury or death myself (*n* = 646)	23 (3.6%)	13 (2%)	15 (2.3%)	592 (91.6%)

Multiple answers were admitted

### Age and education as well as near-death experiences and attacks by family members most significantly affect quality of life

Overall women reported a moderate (overall mean = 3.23, range 1–5, Table 3) quality of life in all domains. Satisfaction with personal relationships ranked highest, while housing conditions led to the greatest dissatisfaction. In comparison to published data from the European Union (EU) region [24], refugee women fared significantly worse overall, yet demonstrated slightly increased satisfaction with personal relationships compared to the EU population. Quality of life, health satisfaction, and satisfaction with living conditions ranked worst in the comparison. For refugee women, sociodemographic factors affect the perceived overall quality of life as well as specific subdomains. Age was a relevant factor in most domains, yet only three remained significant after adjustment in a multivariate model. Younger women were more likely to express satisfaction with their health status (OR for dissatisfaction 0.5, 95% CI 0.4–0.7, *p* < 0.0001, Additional file 5: Table S5) as well as with their overall self-satisfaction (OR for dissatisfaction 0.6, 95% CI 0.4–0.9, *p* = 0.005). Absence of formal schooling positively correlated with the perception of current living conditions (OR for dissatisfaction 0.4, 95% CI 0.3–0.7, *p* < 0.001). We then investigated the impact of traumatic experiences on quality of life perception. Residence and mission in a war zone, near-death experiences, and being sick without any access to health care most significantly affected the different domains of quality of life (Additional file 6: Table S6).

**Table 3:** Comparison between quality of life in the study population and a European reference sample

	Mean FRS	SD FRS	Mean Europe	SD Europe	df	*t*	*p*
How would you rate your quality of life?	2.92	1.05	3.68	0.82	5506	21.55	<0.0001
How satisfied are you with your health?	3.01	1.25	3.6	0.96	5503	14.2	<0.0001
Do you have enough energy for everyday life?	3.42	1.27	3.8	0.91	5505	9.53	<0.0001
How satisfied are you with your ability to perform your daily activities?	3.57	1.28	3.79	0.87	5504	5.7	<0.0001
How satisfied are you with yourself?	3.71	1.33	3.66	0.88	5501	–1.27	0.2
How satisfied are you with your personal relationships?	3.97	1.24	3.89	0.84	5496	–2.14	0.03
Have you enough money to meet your needs?	2.82	1.17	3.14	1.09	5502	6.99	<0.0001
How satisfied are you with the conditions of your living place?	2.47	1.34	3.89	0.97	5504	33.45	<0.0001
Total	3.23	0.78	3.68	0.62	5508	16.9	<0.0001

*FRS* Female Refugee Study, *SD* standard deviation, *df* degrees of freedom

EUROHIS-QOL uses a 5-point Likert scale: very bad (1), bad (2), intermediate (3), good (4), very good (5) [21]

Data for Europe are extracted from Schmidt et al. [24]

We then combined sociodemographic factors and traumatic experiences in unifying models. We sought to identify the most relevant factors affecting the overall quality of life of the interviewed women and specifically their satisfaction with their health and their self-perception. Older age correlated with worse perception of quality of life and greater dissatisfaction with health and self (Table 4). Having had a near-death experience associated with both the overall quality of life and specifically one’s health perception (OR = 1.7, 95% CI = 1.2–2.4, *p* = 0.001 and OR = 2.0, 95% CI = 1.4–2.8, *p* < 0.0001, respectively). Illness with no access to health care also significantly associated with a worse rating of one’s health (OR = 1.7, 95% CI = 1.2–2.6, *p* = 0.004). Attacks by family members correlated with both a worse quality of life and a reduced self-satisfaction. Having had sexual contacts as a minor did not correlate with the overall quality of life, but it significantly associated with a worse self-perception (OR = 2.0, 95% CI = 1.2–3.3, *p* = 0.009).

**Table 4:** Multivariate models of sociodemographic and traumatic variables for reduced quality of life

	OR	95% CI	*p*
a) Quality of life (EUROHIS-QOL, all items), Outcome: mean < 3.23			
Age > 30 years	1.6	1.2–2.3	0.004
Near-death experience	1.7	1.2–2.4	0.001
Mission/residence in war zone	0.7	0.5–1	0.04
Attack by a family member	2	1.3–3.1	0.001
Hosmer-Lemeshow ns, ROC for prediction 0.64			
b) Health satisfaction (item “How satisfied are you with your health?”), Outcome: ranking (very) bad to medium			
Age > 30 years	1.7	1.2–2.4	0.001
Near-death experience	2.0	1.4–2.8	<0.0001
Illness with no access to health care	1.7	1.2–2.6	0.004
Attack by a family member	1.6	1–2.5	0.05
Hosmer-Lemeshow ns, ROC for prediction 0.67			
c) Satisfaction with self (tem “How satisfied are you with yourself?”), Outcome: ranking (very) bad to medium			
Age > 30 years	1.6	1.1–2.2	0.005
Education (no schooling)	0.6	0.4–1	0.04
Attack by a family member	2.5	1.2–3.3	<0.0001
Sexual contacts as a minor	2.0	1.2–3.3	0.009
Hosmer-Lemeshow ns, ROC for prediction 0.64			

*ns* not significant, *ROC* receiver operating characteristic

## Discussion

To our knowledge, the Female Refugee Study (FRS) is the largest study to date documenting the specific situation of refugee women arriving in Germany during the years 2015–2016. The women we interviewed reported a multitude of reasons for flight and experienced trauma in their home countries and during the journey. The vast majority of women resorted to smugglers and described otherwise very limited support during the flight. The interviewed women classify their current quality of life in Germany as average. While they are mostly unsatisfied with their housing conditions, they are generally happy about their relationship situation, displaying differentiated patterns of satisfaction in different domains. Besides the overall quality of life, we were specifically interested in subjective perception of health and self, as these domains appear most relevant for resilience and integration. Both social factors, such as age and education, as well as traumatic experiences, such as near-death situations, attacks by family members, and inability to access health care significantly correlate with quality of life and its subdomains.

The current study offers insight into the life and experiences of refugee women in Germany focusing on different settings and countries of origin. The study design required the selection of women from six nations that maintained a high likelihood of obtaining asylum: Afghanistan, Syria, Iran, Iraq, Somalia, and Eritrea. Women from the Middle East mostly traveled in larger groups with family members and children, while women from eastern Africa reported more frequently traveling alone. This is most likely due to the structural and political situations in their home countries, leading to either mass migration or single journeys escaping a terror regime. We also encountered differential effects of the educational status of the women interviewed, but these cannot be generalized due to differences in the sample sizes depending on the country of origin. The reasons for fleeing were mostly war, terror, and fear for one’s life or the life of family members, demonstrating a high degree of stressors and a perceived unavoidability of migration in many cases. About 25% of the women reported personally witnessing unnatural death or killing of a family member or close friend. Large numbers of women reported homelessness, hunger, and inability to provide for their basic needs.

Gender-specific reasons for fleeing were primarily expressed by women from Afghanistan and Somalia, who most frequently mentioned fear of forced marriage and honor killings. Women of all countries of origin expressed fear of sexual violence as a reason for fleeing, yet fear of genital mutilation was mentioned by 10% of the women from Somalia. This somewhat low number compared to the widespread practice of female genital mutilation (FGM) in the region [25, 26] is surprising. Our study does not address whether FGM is a taboo topic or not considered a relevant reason for fleeing [27], due to either social acceptance, resignation, or the fact that FGM was experienced in childhood and thus does not represent a threat anymore. Twelve percent of the women reported sexual contacts as minors. This might reflect a high incidence of child marriages, but it could also include experiences of sexual coercion. The clear distinction between these two phenomena was beyond the scope of the current study, but it warrants further exploration, especially in consideration of its relevance to self-perception. In fact, having had sexual contacts as a minor negatively correlated with self-satisfaction in our sample. The negative impact of non-consensual sexual actions, especially in minors, on self-respect and self-image has been described before and could be at the root of our observations [28].

Overall, the interviewed women subjectively defined their quality of life as average; however, different patterns in different domains emerged. Satisfaction with personal relationships was elevated, pointing to the role of social support and social cohesion as important factors for well-being [15]. Housing conditions, on the other hand, were the greatest source of disappointment in the population [29] and had a high impact on their quality of life [30]. Women with lower educational status appeared slightly less affected, but the overall perception was predominantly negative. This might directly associate with the conditions of scarcity and overcrowding experienced by the women as well as the lack of privacy and comfort due to shelter conditions in the wake of the 2015 mass migration to Germany. In addition to the direct dissatisfaction with current housing conditions, the apprehension of being unable to secure a private apartment for one’s family in the short and medium term might increase this disappointment [29, 30]. However, housing problems also represent a tangible issue amenable to potential solutions and might have been mentioned more frequently than other relevant problems, which cannot be solved directly, e.g., end of the conflict in the home country or family reunion.

Women older than age 30 were generally inclined to rate their quality of life, health, and self-satisfaction in poorer terms compared to younger women. This might relate to both an objectively worse health status due to the aging process or to subjective perception. Near-death experiences strongly associated with negative health perception and with overall quality of life. These experiences represent lasting trauma in the lives of the affected individuals, as described elsewhere for non-refugee populations [31]. Health perception also significantly correlated with the unavailability of health care in a situation of need. This phenomenon points to a specific area of vulnerability of the women interviewed and is a sensitive topic for service provision in the shelters. Furthermore, the health care provided to asylum seekers in Germany is limited to acute care and does not include psychological counseling [32]. Health access restrictions, hence, can have a double effect on these women: a risk of re-traumatization, since absence of health care was a trauma associated to the experience of war, and a potential worsening of health problems due to absence of care. Finally, attacks by family members not only correlated with overall worse quality of life but most importantly with worse self-perception. Family represents a core protective element and fulfills the basic needs of safety and social belonging; thus, an interference with this elementary safe space can have long-lasting consequences, as described in the literature [33].

Although our study has been designed to include a large group of women from different countries of origin and residing in different areas in Germany, some aspects need to be considered. We recruited the population according to a quota based on the distribution of individuals from the selected countries of origin in Germany. This, however, leads to an overall limited number of women from eastern Africa within the study population, as these individuals make up a small part of the total refugee population in Germany. Hence, the deductions based on the characteristics of these subgroups should be confirmed in larger samples of women from these countries. Furthermore, our recruitment procedure might lead to an over- or underrepresentation of the prevalence of traumatic experiences due to voluntary participation. The current design was selected over a randomized study for both ethical and practical reasons. Ethically, it did not appear acceptable to subject women who have experienced a vast array of traumatic events to a potential re-traumatization based on random allocation; hence, we chose voluntary participation respecting a representative quota. Practically, a large study carried out in the wake of the influx of almost one million people within a host country had to take into account local specificities and logistic constraints. Briefly, this meant, e.g., different regional procedures for ethical approval, different degrees of willingness to support the study depending on the facility management, difficulties in time coordination as the women had to give priority to bureaucratic and health needs over participation in a research study, availability of translators, and other factors, all of which need to be taken into account when comparing our findings to other studies. In order to avoid re-traumatization of the women during the interviews, we avoided intensive questioning if the women refused to respond to some areas of the questionnaire. Although the missing rates were limited in the overall sample, this might lead to a potential underestimation of some forms of trauma. Finally, in order to depict a broad picture of the refugee experience of women fleeing to Europe, we decided to omit more detailed inquiry into certain domains of, e.g., gender-specific violence, which will be addressed in follow-up studies.

## Conclusions

Overall, we offer the first comprehensive description of the flight experience of women from the Middle East and eastern Africa to Germany in the years 2015 and 2016. The investigated population described significant trauma, which correlates with their current quality of life. To identify women at higher risk, we recommend specifically investigating the following four aspects: age, subjective near-death experiences, absence of health care in the case of illness, and having experienced violence by a family member. This should aid health and social workers in identifying those at major risk in order to monitor and support them as needed.

## Additional files

Additional file 1: Table S1. Accompanying persons on flight. (DOCX 12 kb)

Additional file 2: Table S2. Reasons for flight. (DOCX 12 kb)

Additional file 3: Table S3. Supporters on flight. (DOCX 14 kb)

Additional file 4: Table S4. Type of support on flight. (DOCX 14 kb)

Additional file 5: Table S5. Association between sociodemographic variables, quality of life, health perception, and need satisfaction. (DOCX 13 kb)

Additional file 6: Table S6. Multivariate association between quality of life, health perception, need satisfaction, and traumatic experience on or before flight. (DOCX 12 kb)

## Abbreviations

BAMF: Federal Office for Migration and Refugees
FRS: Female Refugee Study
UNHCR: United Nations High Commissioner for Refugees
