# Supplementary material for: Near-death experiences, attacks by family members, and absence of health care in their home countries affect the quality of life of refugee women in Germany: a multi-region, cross-sectional, gender-sensitive study
Source: BMC Med. 2018 Feb 1;16:15. doi: 10.1186/s12916-017-1003-5 (PMC5793395; doi:10.1186/s12916-017-1003-5)
Supplement: Supplementary file 1 — Accompanying persons on flight. (DOCX 12 kb) [file 12916_2017_1003_MOESM1_ESM.docx]

Additional file 1: Table S1. Accompanying persons on flight

|  | **Afghanistan** | **Syria** | **Iraq** | **Somalia** | **Iran** | **Eritrea** |
| --- | --- | --- | --- | --- | --- | --- |
| Nobody | 4 (2.5%) | 16 (5.2%) | 6 (8.2%) | 11 (55%) | 5 (13.2%) | 22 (38.6%) |
| Husband | 116 (71.2%) | 155 (49.8%) | 39 (53.4%) | 2 (10%) | 28 (73.7%) | 6 (10.5%) |
| Partner | 3 (2%) | 0 | 0 | 0 | 1 (2.6%) | 1 (1.8%) |
| Mother | 10 (6.3%) | 21 (6.8%) | 5 (6.9%) | 0 | 1 (2.6%) | 0 |
| Father | 9 (5.5%) | 14 (4.5%) | 1 (1.4%) | 0 | 1 (2.6%) | 0 |
| Children | 111 (68.1%) | 214 (68.8%) | 52 (71.2%) | 2 (10%) | 17 (44.7%) | 14 (24.6%) |
| Sister(s) | 7 (4.3%) | 45 (14.5%) | 5 (6.9%) | 1 (5%) | 1 (2.6%) | 3 (5.3%) |
| Brother(s) | 11 (6.8%) | 29 (9.3%) | 8 (11%) | 0 | 2 (5.3%) | 2 (3.5%) |
| Other relatives | 29 (17.8%) | 85 (27.3%) | 17 (23.3%) | 1 (5%) | 4 (10.5%) | 1 (1.8%) |
| Friends | 6 (3.7%) | 13 (4.2%) | 5 (6.9%) | 1 (5%) | 2 (5.3%) | 2 (3.5%) |
| People met on the journey | 33 (20.3%) | 61 (19.6%) | 8 (11%) | 1 (5%) | 6 (15.8%) | 11 (19.3%) |

Multiple answers were admitted.
