# Supplementary material for: Near-death experiences, attacks by family members, and absence of health care in their home countries affect the quality of life of refugee women in Germany: a multi-region, cross-sectional, gender-sensitive study
Source: BMC Med. 2018 Feb 1;16:15. doi: 10.1186/s12916-017-1003-5 (PMC5793395; doi:10.1186/s12916-017-1003-5)
Supplement: Supplementary file 2 — Reasons for flight. (DOCX 12 kb) [file 12916_2017_1003_MOESM2_ESM.docx]

Additional file 2: Table S2. Reasons for flight

|  | **Afghanistan** | **Syria** | **Iraq** | **Somalia** | **Iran** | **Eritrea** |
| --- | --- | --- | --- | --- | --- | --- |
| Danger to my life / life of my family | 144 (90%) | 256 (82%) | 61 (84%) | 11 (55%) | 21 (55%) | 37 (65%) |
| War | 79 (49%) | 297 (95%) | 60 (82%) | 14 (70%) | 3 (8%) | 17 (30%) |
| Terror regime | 65 (41%) | 242 (78%) | 62 (85%) | 8 (40%) | 2 (5%) | 13 (23%) |
| Threat of torture to me/my family | 85 (53%) | 60 (19%) | 33 (45%) | 7 (35%) | 12 (32%) | 22 (39%) |
| Experienced gender-based violence | 57 (36%) | 35 (11%) | 15 (21%) | 7 (35%) | 10 (26%) | 13 (23%) |
| Fear of sexual violence | 46 (29%) | 84 (27%) | 12 (16%) | 6 (30%) | 6 (16%) | 9 (16%) |
| Fear of honor killing | 65 (41%) | 64 (21%) | 17 (23%) | 10 (50%) | 10 (26%) | 7 (12%) |
| Fear of forced marriage | 32 (20%) | 10 (3%) | 6 (8%) | 7 (35%) | 5 (13%) | 0 |
| Fear of genital mutilation for me/my children | 2 (1%) | 15 (5%) | 4 (5%) | 2 (10%) | 0 | 2 (4%) |
| Fear of abduction | 59 (37%) | 139 (45%) | 39 (53%) | 4 (20%) | 6 (16%) | 6 (11%) |
| Religious prosecution | 17 (11%) | 41 (13%) | 31 (42%) | 1 (5%) | 17 (45%) | 6 (11%) |
| Prosecution due to sexual identity or orientation | 1 (1%) | 17 (5%) | 6 (8%) | 0 | 1 (3%) | 0 |
| Prosecution due to political activity | 12 (8%) | 23 (7%) | 3 (4%) | 1 (5%) | 9 (24%) | 12 (21%) |
| My family already in Germany | 9 (6%) | 64 (21%) | 8 (11%) | 0 | 3 (8%) | 1 (2%) |
| Incumbent military duty for me / my partner | 14 (9%) | 73 (23%) | 4 (5%) | 1 (5%) | 2 (5%) | 13 (23%) |
| Inability to secure elementary needs | 14 (9%) | 185 (59%) | 38 (52%) | 7 (35%) | 4 (11%) | 6 (11%) |
| Not enough food | 6 (4%) | 130 (42%) | 20 (27%) | 6 (30%) | 1 (3%) | 3 (5%) |

Multiple answers were admitted.
