# Supplementary material for: Near-death experiences, attacks by family members, and absence of health care in their home countries affect the quality of life of refugee women in Germany: a multi-region, cross-sectional, gender-sensitive study
Source: BMC Med. 2018 Feb 1;16:15. doi: 10.1186/s12916-017-1003-5 (PMC5793395; doi:10.1186/s12916-017-1003-5)
Supplement: Supplementary file 3 — Supporters on flight. (DOCX 14 kb) [file 12916_2017_1003_MOESM3_ESM.docx]

Additional file 3: Table S3. Supporters on flight

|  | **Afghanistan** | **Syria** | **Iraq** | **Somalia** | **Iran** | **Eritrea** |
| --- | --- | --- | --- | --- | --- | --- |
| Fellow travelers | 39 (24%) | 107 (34%) | 13 (18%) | 4 (20%) | 8 (21%) | 24 (42%) |
| Private persons | 27 (17%) | 50 (16%) | 16 (22%) | 6 (30%) | 4 (11%) | 10 (18%) |
| Volunteers | 20 (13%) | 38 (12%) | 9 (12%) | 0 | 4 (11%) | 0 |
| Governmental organizations | 41 (25%) | 48 (15%) | 15 (21%) | 0 | 6 (16%) | 2 (4%) |
| Non-governmental organizations | 10 (6%) | 103 (33%) | 19 (26%) | 1 (5%) | 1 (3%) | 4 (7%) |
| Religious institutions | 1 (1%) | 1 (0%) | 0 | 0 | 1 (3%) | 0 |
| Military | 7 (4%) | 3 (1%) | 5 (7%) | 0 | 1 (3%) | 1 (2%) |
| Police | 26 (16%) | 28 (9%) | 8 (11%) | 1 (5%) | 4 (11%) | 0 |
| Others | 16 (10%) | 21 (7%) | 2 (3%) | 7 (35%) | 2 (5%) | 5 (9%) |

Multiple answers were admitted.
