# Supplementary material for: Near-death experiences, attacks by family members, and absence of health care in their home countries affect the quality of life of refugee women in Germany: a multi-region, cross-sectional, gender-sensitive study
Source: BMC Med. 2018 Feb 1;16:15. doi: 10.1186/s12916-017-1003-5 (PMC5793395; doi:10.1186/s12916-017-1003-5)
Supplement: Supplementary file 4 — Type of support on flight. (DOCX 14 kb) [file 12916_2017_1003_MOESM4_ESM.docx]

Additional file 4: Table S4. Type of support on flight

|  | **Afghanistan** | **Syria** | **Iraq** | **Somalia** | **Iran** | **Eritrea** |
| --- | --- | --- | --- | --- | --- | --- |
| Food / drink | 54 (33%) | 165 (53%) | 34 (47%) | 2 (10%) | 12 (32%) | 8 (14%) |
| Clothing | 44 (27%) | 137 (44%) | 27 (37%) | 2 (10%) | 8 (21%) | 5 (9%) |
| Electricity | 11 (7%) | 34 (11%) | 2 (3%) | 0 | 5 (13%) | 0 |
| Orientation | 31 (19%) | 84 (27%) | 16 (22%) | 1 (5%) | 5 (13%) | 1 (2%) |
| Shelter | 33 (20%) | 74 (24%) | 7 (10%) | 2 (10%) | 4 (11%) | 4 (7%) |
| Medical care | 23 (14%) | 91 (29%) | 16 (22%) | 1 (5%) | 6 (16%) | 0 |
| Support/encouragement | 32 (20%) | 36 (12%) | 6 (8%) | 1 (5%) | 1 (3%) | 9 (16%) |
| Use of sanitary facilities | 33 (20%) | 85 (27%) | 14 (19%) | 1 (5%) | 5 (13%) | 0 |
| Transportation | 18 (11%) | 120 (39%) | 24 (33%) | 5 (25%) | 3 (8%) | 0 |

Multiple answers were admitted.
