# Supplementary material for: Near-death experiences, attacks by family members, and absence of health care in their home countries affect the quality of life of refugee women in Germany: a multi-region, cross-sectional, gender-sensitive study
Source: BMC Med. 2018 Feb 1;16:15. doi: 10.1186/s12916-017-1003-5 (PMC5793395; doi:10.1186/s12916-017-1003-5)
Supplement: Supplementary file 5 — Association between sociodemographic variables, quality of life, health perception, and need satisfaction. (DOCX 13 kb) [file 12916_2017_1003_MOESM5_ESM.docx]

|  | **Age^1^** | **Family status^2^** | **Children^3^** | **Schooling^4^** | **Employment^5^** |
| --- | --- | --- | --- | --- | --- |
| *How would you rate your quality of life?* | 0.8 (0.6-1.2)  p=.3 | 1 (0.6-1.6)  p=1 | 1 (0.6-1.6)  p=1 | 0.8 (0.5-1.2)  p=.2 | 0.9 (0.7-1.4)  p=.8 |
| *How satisfied are you with your health?* | 0.5 (0.4-0.8)  **p<.0001** | 1.2 (0.8-1.8)  p=.4 | 1.3 (0.8-2.1)  p=.3 | 1 (0.7-1.6)  p=.9 | 0.8 (0.6-1.2)  p=.3 |
| *Do you have enough energy for everyday life?* | 0.7 (0.5-0.9)  **p=.02** | 1 (0.6-1.5)  p=.9 | 0.8 (0.5-1.3)  p=.5 | 1.1 (0.7-1.7)  p=.7 | 0.8 (0.6-1.1)  p=.2 |
| *How satisfied are you with your ability to perform your daily activities?* | 0.7 (0.5-1)  p=.07 | 1.1 (0.8-1.7)  p=.5 | 0.7 (0.5-1.1)  p=.2 | 1 (0.7-1.6)  p=1 | 1 (0.7-1.3)  p=.8 |
| *How satisfied are you with yourself?* | 0.6 (0.4-0.9)  **p=.005** | 0.9 (0.6-1.3)  p=.5 | 1 (0.6-1.6)  p=1 | 0.7 (0.4-1.1)  p=.09 | 1 (0.7-1.4)  p=.9 |
| *How satisfied are you with your personal relationships?* | 0.9 (0.6-1.3)  p=.7 | 1.1 (0.7-1.7)  p=.6 | 0.9 (0.6-1.5)  p=.7 | 0.8 (0.5-1.3)  p=.3 | 0.9 (0.6-1.2)  p=.4 |
| *Have you enough money to meet your needs?* | 1 (0.7-1.4)  p=1 | 1.3 (0.8-2.2)  p=.3 | 1.2 (0.7-2)  p=.5 | 0.8 (0.5-1.3)  p=.4 | 0.9 (0.6-1.3)  p=.6 |
| *How satisfied are you with the conditions of your living place?* | 1.4 (0.9-2.1)  p=.1 | 0.8 (0.5-1.2)  p=.3 | 1.1 (0.6-2)  p=.7 | 0.4 (0.3-0.7)  **p<.0001** | 0.9 (0.6-1.4)  p=.8 |

Additional file 5: Table S5. Association between sociodemographic variables, quality of life, health perception and need satisfaction.

^1^Age below or above 30 years; reference: age <30 years

^2^Partnered/married versus traveling alone (single, divorced, widowed); reference: alone

^3^Having own children or not having any; reference: no children

^4^No school attendance versus any school attendance; reference: no schooling

^5^Having had a formal employment in the home country versus being a homemaker/no employment; reference: having had employment

^§^N=637 (questionnaires were included if at least 6 of the 8 items were answered)
