# Supplementary material for: Near-death experiences, attacks by family members, and absence of health care in their home countries affect the quality of life of refugee women in Germany: a multi-region, cross-sectional, gender-sensitive study
Source: BMC Med. 2018 Feb 1;16:15. doi: 10.1186/s12916-017-1003-5 (PMC5793395; doi:10.1186/s12916-017-1003-5)
Supplement: Supplementary file 6 — Multivariate association between quality of life, health perception, need satisfaction, and traumatic experience on or before flight. (DOCX 12 kb) [file 12916_2017_1003_MOESM6_ESM.docx]

|  | **Mission/residence in war zone** | **Near death experience** | **Sick with no access to healthcare** | **Aggression from family member** | **Forced isolation** |
| --- | --- | --- | --- | --- | --- |
| *How would you rate your quality of life?* | 0.6 (0.4-0.9)  ***p*= .02** | 1.5 (1-2.3)  ***p*= .05** | 1.4 (0.9-2)  *p*=.1 | 1.4 (0.8-2.4)  *p*=.2 | 0.5 (0.3-0.8)  ***p*=.008** |
| *How satisfied are you with your health?* | 0.7 (0.5-1)  *p*=.08 | 2.3 (1.5-3.3)  ***p*<.0001** | 1.7 (1.1-2.4)  ***p*=.008** | 1.7 (1.1-2.8)  ***p*=.02** | 0.9 (0.5-1.5)  *p*=.7 |
| *Do you have enough energy for everyday life?* | 0.8 (0.6-1.1)  *p*=.2 | 1.4 (1-2)  *p*=.06 | 1.3 (0.9-1.9)  *p*=.1 | 1.4 (0.9-2.2)  *p*=.1 | 0.7 (0.5 -1.2)  *p*=.2 |
| *How satisfied are you with your ability to perform your daily activities?* | 0.8 (0.5-1)  ***p*=.01** | 1.5 (1-2.1)  ***p*=.04** | 1.5 (1-2.1)  ***p*=.04** | 1.2 (0.8-1.9)  *p*=.3 | 0.6 (0.4-1)  ***p*=.05** |
| *How satisfied are you with yourself?* | 0.8 (0.6-1.1)  *p*=.2 | 1.3 (0.9-1.8)  *p*= .2 | 1.1 (0.8-1.6)  *p*=.6 | 2.4 (1.5-3.7)  ***p*<.0001** | 1.3 (0.8-2.2)  *p*=.2 |
| *How satisfied are you with your personal relationships?* | 0.6 (0.4-0.9)  ***p*=.01** | 1.3 (0.9-1.9)  *p*=.2 | 1.5 (1-2.1)  ***p*=.03** | 1.7 (1.1-2.6)  ***p*=.03** | 0.9 (0.5-1.4)  *p*=.6 |
| *Have you enough money to meet your needs?* | 0.5 (0.4-0.8)  ***P*=.001** | 1.3 (0.8-2)  *p*=.2 | 1.6 (1-2.4)  ***p*=.04** | 1.4 (0.8-2.3)  *p*=.2 | 1.4 (0.8-2.6)  *p*=.2 |
| *How satisfied are you with the conditions of your living place?* | 0.6 (0.4-0.9)  ***p*=.01** | 1.4 (0.9-2.2)  *p*=.1 | 1.5 (0.9-2.3)  *p*=.09 | 1 (0.6-1.7)  *p*=.9 | 0.4 (0.3-0.8)  ***p*=.003** |

Additional file 6: Table S6. Multivariate association between quality of life, health perception, need satisfaction and traumatic experience on or before flight.

Traumatic experience variables were adjusted for all trauma experience (see table 2). Multiple answers were possible.

Only variables displaying significant associations are reported in the current table and were included in the final models.
